# Retrosplenial Cortex Effects Contextual Fear Formation Relying on Dysgranular Constituent in Rats

**DOI:** 10.3389/fnins.2022.886858

**Published:** 2022-05-03

**Authors:** Ting-Ting Pan, Chao Liu, De-Min Li, Tian-Hao Zhang, Wei Zhang, Shi-Lun Zhao, Qi-Xin Zhou, Bin-Bin Nie, Gao-Hong Zhu, Lin Xu, Hua Liu

**Affiliations:** ^1^School of Physics and Microelectronics, Zhengzhou University, Zhengzhou, China; ^2^Beijing Engineering Research Center of Radiographic Techniques and Equipment, Institute of High Energy Physics, Chinese Academy of Sciences, Beijing, China; ^3^CAS Key Laboratory of Animal Models and Human Disease Mechanisms, and KIZ-SU Joint Laboratory of Animal Model and Drug Development, and Laboratory of Learning and Memory, Kunming Institute of Zoology, Chinese Academy of Sciences, Kunming, China; ^4^Kunming College of Life Science, University of Chinese Academy of Sciences, Kunming, China; ^5^School of Nuclear Science and Technology, University of Chinese Academy of Sciences, Beijing, China; ^6^Department of Nuclear Medicine, The First Affiliated Hospital of Kunming Medical University, Kunming, China; ^7^CAS Centre for Excellence in Brain Science and Intelligent Technology, Shanghai, China

**Keywords:** contextual fear formation, retrosplenial cortex, brain metabolic network, graph theory, hippocampal-amygdalar system

## Abstract

Animal contextual fear conditioning (CFC) models are the most-studied forms used to explore the neural substances of posttraumatic stress disorder (PTSD). In addition to the well-recognized hippocampal–amygdalar system, the retrosplenial cortex (RSC) is getting more and more attention due to substantial involvement in CFC, but with a poor understanding of the specific roles of its two major constituents—dysgranular (RSCd) and granular (RSCg). The current study sought to identify their roles and underlying brain network mechanisms during the encoding processing of the rat CFC model. Rats with pharmacologically inactivated RSCd, RSCg, and corresponding controls underwent contextual fear conditioning. [^18^F]-fluorodeoxyglucose positron emission tomography/computed tomography (^18^F-FDG PET/CT) scanning was performed for each animal. The 5-h and 24-h retrieval were followed to test the formation of contextual memory. Graph theoretic tools were used to identify the brain metabolic network involved in encoding phase, and changes of nodal (brain region) properties linked, respectively, to disturbed RSCd and RSCg were analyzed. Impaired retrieval occurred in disturbed RSCd animals, not in RSCg ones. The RSC, hippocampus (Hip), amygdala (Amy), piriform cortex (Pir), and visual cortex (VC) are hub nodes of the brain-wide network for contextual fear memory encoding in rats. Nodal degree and efficiency of hippocampus and its connectivity with amygdala, Pir, and VC were decreased in rats with disturbed RSCd, while not in those with suppressed RSCg. The RSC plays its role in contextual fear memory encoding mainly relying on its RSCd part, whose condition would influence the activity of the hippocampal–amygdalar system.

## Introduction

The posttraumatic stress disorder (PTSD) is a kind of anxiety disorder usually occurring after undergoing a traumatic event, of which the neural mechanism remains unclear (Brunello et al., 2001; Keane et al., 2006). Animal contextual fear conditioning (CFC) models are the most-studied forms for understanding PTSD and other stress-related disorders (Izquierdo et al., 2016). Defining the key neural correlates and circuitries involved in CFC is fundamental for understanding its underlying mechanism. Although the hippocampus (Hip), amygdala (Amy) nuclei, and the forebrain cortex are popularly recognized (Izquierdo et al., 2016; Alexandra Kredlow et al., 2022), a comprehensive understanding of the key brain regions and their interactions linked to CFC is still in the face of many challenges. Recently, the retrosplenial cortex (RSC) has received more and more attention because of its general activation in episodic memory (Vann et al., 2009; Corcoran et al., 2018).

Early evidence came from RSC lesion studies in animals showing the RSC was necessary in associative learning situations such as eyeblink conditioning and discrimination reversal learning (Gabriel et al., 1983; Berger et al., 1986). Later, in addition to spatial processing and navigation (Cooper and Mizumori, 2001; Vann and Aggleton, 2002), RSC was also found substantially involved in instrumental and associative learning of aversive stimuli (Lukoyanov and Lukoyanova, 2006; Keene and Bucci, 2008). Recent works exploring interactions between the hippocampus and other cortices in contextual fear memory demonstrated that the RSC was always engaged in, ranging from the encoding and retrieval phases to extinction (Cowansage et al., 2014; Jovasevic et al., 2015; Leaderbrand et al., 2016). Human imaging studies showed increased activity of RSC during contextual memory retrieval and autobiographical memory in healthy and PTSD subjects (Liberzon et al., 1999; Schacter and Addis, 2007). Furthermore, some neurodegenerative disorders with amnesia, typically Alzheimer’s disease, often accompany RSC pathological changes (Leech and Sharp, 2014; Weiner et al., 2017). Although numerous studies have suggested in principle the involvement of RSC in fear memory, the issue of its structural heterogeneity should be considered. Cytoarchitecturally, the RSC is composed of two major parts, granular area (RSCg) and dysgranular area (RSCd) (Vann et al., 2009). These two subregions are different in structural connections with other regions (Vogt and Miller, 1983; Vogt et al., 2005), which implies that they likely play distinct roles functionally. A few pilot studies exhibited RSCg and RSCd played different roles in spatial cognitive tasks (Pothuizen et al., 2009), supporting the view of functional heterogeneity of RSC. In contextual fear memory, however, the individual roles of RSCg and RSCd still remain unknown.

The brain is a complex system with glucose as the main energy source (Mergenthaler et al., 2013), and ^18^F-FDG PET imaging is widely used to study neural activities based on glucose metabolism. Relative to simple tests for regional metabolic patterns due to physiologic and pathologic activity, FDG metabolic brain networks would evaluate the property changes of the system globally as well as the relationships among local brain regions at the whole brain level, which is a significant advantage of this method to reveal the mechanisms of complex systems (Yakushev et al., 2017). Nowadays, metabolic brain networks based on FDG-PET images have been emerging as a useful tool in basic and clinical neuroscience (Zhang et al., 2019; Huang et al., 2020). In this study, to determine the roles of RSCg and RSCd during CFC of rat model, combining pharmacological approaches with [^18^F]-fluorodeoxyglucose positron emission tomography/computed tomography (^18^F-FDG PET/CT) imaging and brain network methods, we investigated their effects and the underlying network mechanisms.

## Materials and Methods

### Animals

Nine-week-old male Sprague-Dawley rats obtained from Vital River Laboratory Animal Technology Company (Beijing, China) were used in this study. The metabolic brain networks were generally constructed by Pearson’s correlations in an intersubject manner. Based on considerations of statistical power, a large sample size was always employed. In this study, four independent groups and a total of 113 rats were involved. Rats were initially housed in groups (five rats per cage) and then housed individually after surgery. Rats had free access to food and water under a 12-h light/12-h dark cycle in a temperature-regulated environment maintained at 25 ± 2°C at the Kunming Institute of Zoology (CAS, Kunming, China). All protocols were approved by the Institutional Animal Care and Use Committee of Kunming Institute of Zoology, Chinese Academy of Sciences (ID: SMKX-20190820-195).

### Surgery and Infusions

Rats had surgery to intracranial implantation stainless steel guide cannula (RWD Life Sciences, Shenzhen, China), using the techniques described previously (Zhou et al., 2017). The stereotaxic coordinates of RSCd (-3 mm posterior, ± 0.6 mm lateral, 1 mm ventral to bregma) and RSCg (-5.20 mm posterior, ± 0.4 mm lateral, 2 mm ventral to bregma) were determined according to the Paxinos and Watson brain atlas (Supplementary Figure 1; Paxinos and Watson, 2005). Rats were allowed 7 days to recover from surgery before the behavioral procedures.

Tetrodotoxin (TTX, 20 μM, T111387-1 mg, Aladdin, Shanghai, China), the sodium channel blocker, was used as an inhibitor to inactivate the target regions in this study (Zhou et al., 2017); saline was used as vehicle. The inhibitor was infused at a volume of 1 μl per side into the target brain regions through the implanted guide cannulas by using the injection needles connecting to a syringe pump (LSP02-2B, Longer Precision Pump Co., Ltd., Baoding, China), and infused at a speed of 0.1 μl/min. All inhibitor and vehicle infusions were made 30 min before placing the rat in the conditioning chamber for training.

### Contextual Fear Conditioning and Test

**FIGURE 1:**
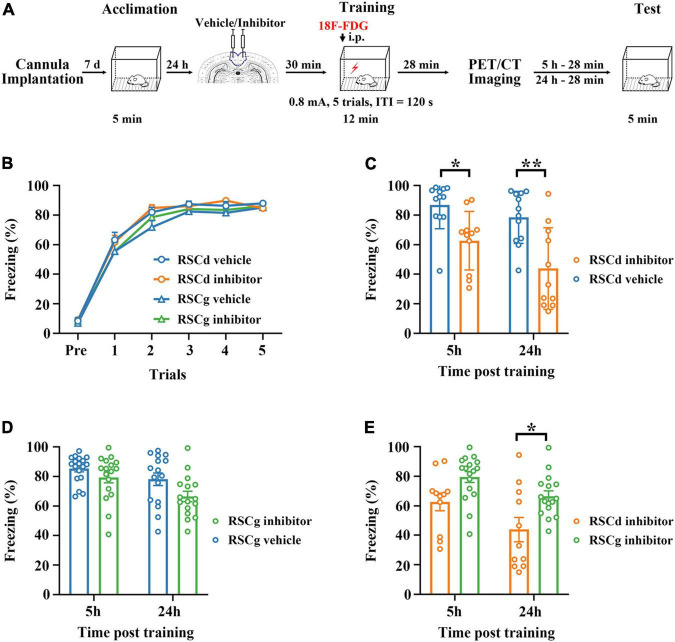
Effects of suppressed RSCd/g on contextual fear memory formation. **(A)** Diagram for experimental procedures. **(B)** The learning curves of four group rats (RSCd vehicle/inhibitor group and RSCg vehicle/inhibitor group) show no significant difference; RSCd vehicle group *n* = 24, inhibitor group *n* = 22; RSCg vehicle group *n* = 35, inhibitor group *n* = 32. **(C)** Memory retrieval during the 5-h and 24-h post-learning tests are significantly impaired in the RSCd inhibitor independent groups compared to the RSCd vehicle independent groups; 5 h, RSCd vehicle group *n* = 12, inhibitor group *n* = 11; 24 h, RSCd vehicle group *n* = 12, inhibitor group *n* = 11. **(D)** There is no significant difference in memory retrieval between the RSCg inhibitor independent groups during the 5-h and 24-h post-learning tests compared to the RSCg vehicle independent groups; 5 h, RSCg vehicle group *n* = 18, inhibitor group *n* = 16; 24 h, RSCg vehicle group *n* = 17, inhibitor group *n* = 16. **(E)** Memory retrieval during the 5-h and 24-h post-learning is significantly impaired in the RSCd inhibitor groups compared to the RSCg inhibitor groups; 5 h, RSCd inhibitor group *n* = 11, RSCg inhibitor group *n* = 16; 24 h, RSCd inhibitor group *n* = 11, RSCg inhibitor group *n* = 16. ITI, intertrial interval; i.p., intraperitoneal injection. *p < 0.05, **p < 0.01, two-way ANOVA followed by Bonferroni’s posttests.

The procedures for CFC and retrieval were shown schematically in Figure 1A (Zhou et al., 2017). One day before CFC, rats were placed in the training box (MED-VFC-SCT-R, MED Associates Inc., Fairfax, VT, United States) to acclimate to the box by exploring for 12 min. On the day of training, the rats were placed in the training box and received 5 footshocks (0.8 mA, 2 s duration) with 2 min intertrial interval, and then returned to the homecage 2 min after final footshock. Retrieval tests were performed by placing the rats in the training box for 5 min without footshocks for independent groups at 5 or 24 h after conditioning. Freezing levels were recorded for scoring fear memory through the computer system (MED-VFC-SCT-R, MED Associates Inc., Fairfax, VA, United States).

### ^18^F-FDG PET/CT Imaging Protocols and Image Preprocessing

Thirty minutes after the inhibitor (or vehicle) injection into the target brain area, rats were injected intraperitoneally with ^18^F-FDG (18.5 MBq/100 g of body weight). Then each rat was immediately subjected to fear training; 40 min after FDG injection, rats were anesthetized with isoflurane (5% for induction and 1.5–2% for maintenance) and fixed in a prone position on the scanning bed of an E-plus 166 micro-PET/CT scanner (Institute of High Energy Physics, CAS, Beijing, China). The scanning was performed for 20 min. After acquisition, the PET images were reconstructed by the two-dimensional ordered subset expectation maximization algorithm with corrections for decay, normalization, dead time, photon attenuation, scatter, and random coincidences, and the reconstructed image matrix size was 256 × 256 × 63 with voxel size of 0.5mm × 0.5mm × 1mm (Nie et al., 2014).

All the images were preprocessed using an improved toolbox for voxel-wise analysis of rat brain images based on SPM8 (Wellcome Department of Cognitive Neurology, London, United Kingdom) (Nie et al., 2014). The images preprocessing steps were as follows: (1) the individual images of rat were spatially normalized into Paxinos and Watson space; (2) the normalized images were smoothened with 2mm × 2mm × 4 mm Gaussian kernel; and (3) then the intensity of images was globally normalized in each image.

### Construction of Brain-Wide Metabolic Network During Contextual Fear Conditioning in Rats

The metabolic networks were constructed as a collection of 39 nodes representing brain regions connected by edges corresponding to the links between them; 39 anatomical brain regions (Supplementary Figure 2 and Supplementary Table 1) were predefined by a three-dimensional digital map based on the Paxinos and Watson atlas (Nie et al., 2013). The correlation coefficient between each pair of nodes was calculated with Pearson’s correlation in an intersubject manner, and the absolute value of correlation coefficient was used to define the weight of the edge (Zhang et al., 2019).

### Hub Nodes and Nodal Properties

Graph theoretic measurements were used to analyze the node properties. In this study, we mainly focused on the nodal degree and nodal efficiency, which characterized the node’s connectivity and efficiency of parallel information transfer in the network, respectively (Choi et al., 2014; Zhang et al., 2019).

The nodal degree is the sum weight that links a node to the rest of the network in a weighted network. We quantitatively determined the hubs by the nodal *Z*-score. *Z*_*i*_-score is defined to measure how well-connected node *i* is to nodes in a network (Zhang et al., 2019), which makes the nodal degree comparable between networks. The *Z*_*i*_-score is defined as


$${\text{Z}}_{\text{i}}=\frac{k_{i}-\bar{k}}{\sigma_{k}},$$

where _*k_i_=∑_j≠i_ A_ij_δ(i,j)*_ is the sum of the weight between node *i* and other nodes in a weighted network. ${}_{\bar{k}}$ is the average of _*k*_ over all the nodes, and _σ_k__ is the standard deviation of _*k*_ in a weighted network. Node *i* is defined as a hub when *Z*_*i*_-score > 1 (Zhang et al., 2019).

Nodal efficiency (E_*nodal*_) characterizes the efficiency of this node’s parallel information transfer in the network. The E_*nodal*_ for node *i* is defined as


$${\text{E}}_{\text{nodal}}\left(i\right)=\frac{1}{N-1}\sum_{i\neq j}\frac{1}{L_{i,j}},$$

where _*L_ij_*_ is the minimum path length between nodes *i*, *j*, and *N* is the number of nodes in a graph (Choi et al., 2014).

### Core Brain Regions Associated With RSCd/g Metabolism During Contextual Fear Conditioning

Based on the brain-wide metabolic network of vehicle group, RSCd and RSCg were, respectively, selected as seed regions to search for brain regions highly metabolically relevant to them to construct their core network during CFC. Pearson’s correlation coefficients were calculated of the mean image intensity between the 39 nodes and the seed region. A strong Pearson’s correlation coefficient of 0.7 was used as a threshold to screen the brain regions that were strongly correlated with the seed region, and the supra-threshold brain regions were considered to form the core metabolic network related to RSCd/RSCg during CFC.

### Statistical Analysis

Two-way ANOVA followed by Bonferroni’s posttests for multiple comparisons was used to analyze parameters of behavior performance. The value of *p* < 0.05 was considered statistically significant. Data were given as mean ± SEM. The permutation tests were used to evaluate statistical differences in node properties. First, the node properties of the metabolic networks were calculated separately for the inhibitor group and the corresponding vehicle group. Then the FDG images of the rat brain were randomly reallocated in two groups, and the correlation matrices for each randomized group were computed. The differences in the node properties between the two randomized networks were then calculated. The permutation tests were also used to evaluate the statistical differences in connection strengths between pairs of nodes in different networks. Permutations were repeated 10,000 times, and the results were used to estimate 95% confidence interval differences.

## Results

### Suppressing RSCd During Fear Conditioning Impaired Memory Formation but Not While Suppressing RSCg

We examined the formation of fear memory under different treatments for independent groups, which were RSCd vehicle group, RSCd inhibitor group, RSCg vehicle group, and RSCg inhibitior group. As shown in Figure 1B, the freezing of rats was gradually formed as the number of training trials increased, and different vehicle/inhibitor treatment did not cause intergroup differences [Figure 1B: RSCd vehicle *n* = 24, inhibitor *n* = 22; RSCg vehicle *n* = 35, inhibitor *n* = 32; trials, *F*_(5, 545)_ = 561.0, *p* < 0.001; group, *F*_(3, 545)_ = 1.300, *p* = 0.278; trials × group, *F*_(15, 545)_ = 0.806, *p* = 0.671; two-way ANOVA with repeated measures followed by Bonferroni’s posttests]. Memory retrieval at 5 or 24 h after learning for the independent groups of RSCd inhibitor was impaired to be more severe compared to RSCd vehicle groups [Figure 1C, 5 h: RSCd vehicle *n* = 12, inhibitor *n* = 11; 24 h: RSCd vehicle *n* = 12, inhibitor *n* = 11; time, *F*_(1, 42)_ = 5.003, *p* < 0.05; group, *F*_(1, 42)_ = 23.410, *p* < 0.001; time × group, *F*_(1, 42)_ = 0.739, *p* = 0.3949; *post hoc*: 5 h: vehicle vs. inhibitor, *p < 0.05; 24 h: vehicle vs. inhibitor, ^**^p < 0.001; two-way ANOVA followed by Bonferroni’s posttests]. Similarly, for RSCg independent groups, both time and inhibitor treatment provoked significant impairment in memory retrieval. But for the same time point, inhibitor treatment did not induce significant impairment of memory [Figure 1D, 5 h: RSCg vehicle *n* = 18, inhibitor *n* = 17; 24 h: RSCg vehicle *n* = 16, inhibitor *n* = 16; time, *F*_(1, 63)_ = 8.377, *p* < 0.01; group, *F*_(1, 63)_ = 6.424, *p* < 0.05; time × group, *F*_(1, 63)_ = 0.709, *p* = 0.403; *post hoc*: 5 h: vehicle vs. inhibitor, *p* = 0.784; 24 h: vehicle vs. inhibitor, *p* = 0.129; two-way ANOVA followed by Bonferroni’s posttests]. In addition, we contrasted the effects of inhibiting the two subregions; memory retrieval at 5 and 24 h after learning for RSCd inhibitor groups were impaired to be more severe over time compared to RSCg inhibitor groups [Figure 1E, time, *F*_(1, 51)_ = 9.220, *p* < 0.01; region, *F*_(1, 51)_ = 14.330, *p* < 0.001; time × region, *F*_(1, 51)_ = 0.317, *p* = 0.576; *post hoc*: 5 h: RSCd vs. RSCg, *p* = 0.147; 24 h: RSCd vs. RSCg, *p < 0.05; two-way ANOVA followed by Bonferroni’s posttests]. This seems to indicate that, respectively, suppressing the two subregions of the RSC before encoding does not interfere with the formation of the contextual fear memory, but suppressing RSCd would produce a profound disruption in the memory retrieval, whereas suppressing RSCg has no such effect.

### Effects on Hub Nodes of the Brain-Wide Metabolic Brain Network of Suppressed RSCd/g During Contextual Fear Conditioning

**FIGURE 2:**
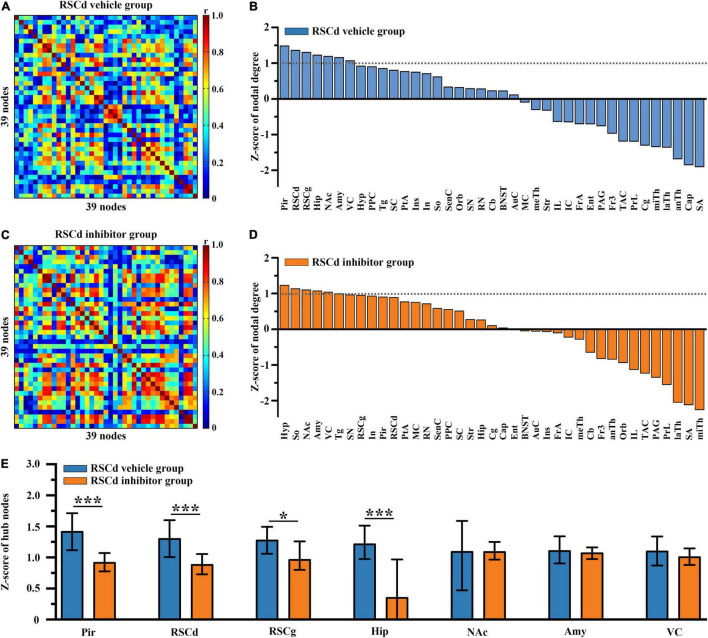
Effects of suppressed RSCd on hub nodes of the brain-wide metabolic network during CFC. Brain-wide metabolic network of **(A)** RSCd vehicle group and **(C)** inhibitor group during CFC. Distribution of nodal degree Z-scores in network of **(B)** RSCd vehicle group and **(D)** inhibitor group. **(E)** Decrease in nodal degree of Pir, RSCd, RSCg, and hippocampus in rats with suppressed RSCd. The error bar indicates the 95% confidence intervals obtained from 1,000-iteration bootstrapping procedures. RSCd vehicle group *n* = 24, RSCd inhibitor group *n* = 22. *p < 0.05, ***p < 0.001, permutation test.

**FIGURE 3:**
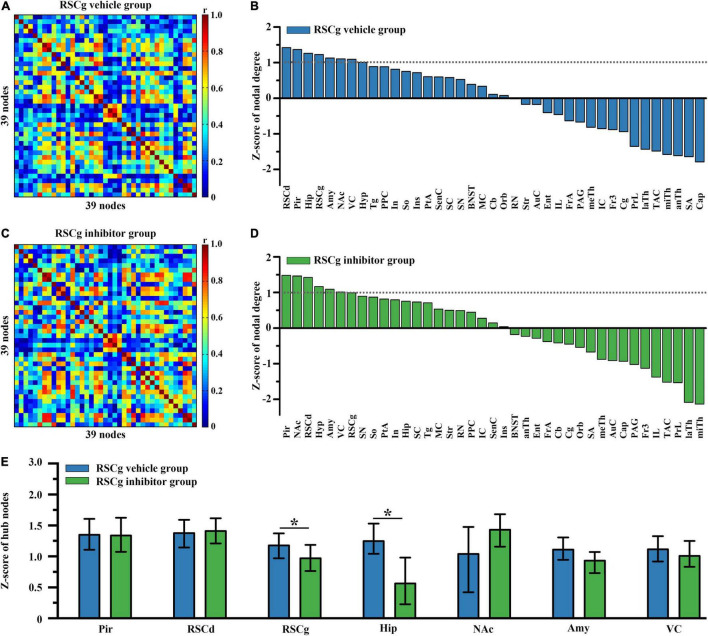
Effects of suppressed RSCg on hub nodes of the brain-wide metabolic network during CFC. Brain-wide metabolic network of **(A)** RSCg vehicle group and **(C)** inhibitor group during CFC. Distribution of nodal degree *Z*-scores in network of **(B)** RSCg vehicle and **(D)** inhibitor group. **(E)** Decrease in nodal degree of RSCg and hippocampus in rats with suppressed RSCg. The error bar indicates the 95% confidence intervals obtained from 1,000-iteration bootstrapping procedures. RSCg vehicle group *n* = 35, RSCg inhibitor group *n* = 32. *p < 0.05, permutation test.

In order to examine the differences in hub nodes of brain networks between independent groups under different treatments, we, respectively, constructed the brain-wide metabolic network during CFC for RSCd vehicle/inhibitor group (Figures 2A,C) and RSCg vehicle/inhibitor group (Figures 3A,C). The analysis of network nodal degree revealed that the hub nodes of brain-wide metabolic network during CFC included RSCd, piriform cortex (Pir), hippocampus, RSCg, amygdala, nucleus accumbens (NAc), and visual cortex (VC) (Figures 2B, 3B and Supplementary Table 2). In the RSCd group, *Z*-score of the nodal degree of hippocampus, Pir, RSCg, RSCd, and amygdala significantly decreased in the inhibitor group (*p < 0.05, ^***^p < 0.001, permutation test; Figures 2D,E). In the RSCg group, Z-score of the nodal degree of hippocampus and RSCg significantly decreased (*p < 0.05, permutation test; Figures 3D,E).

### Effects on Nodal Properties of the Core Network of Suppressed RSCd/g During Contextual Fear Conditioning

**FIGURE 4:**
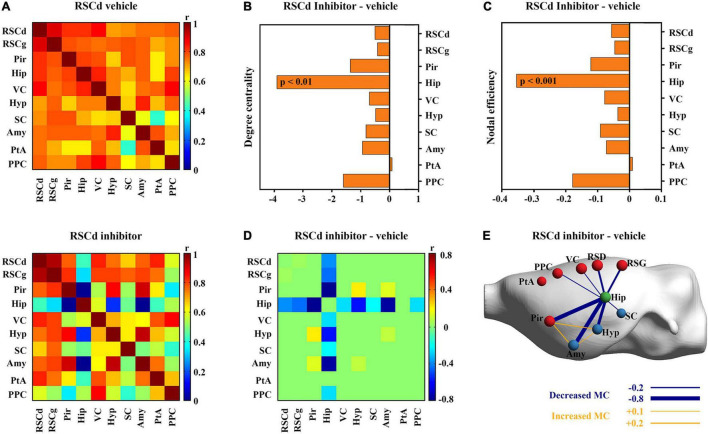
Effects of suppressed RSCd on its core metabolic networks during CFC. **(A)** Core metabolic network of RSCd vehicle group and inhibitor groups during CFC. **(B)** Degree centrality and **(C)** nodal efficiency of the hippocampus significantly decrease in rats with suppressed RSCd. **(D)** 10,000 permutation tests reveal a significant decrease in metabolic correlations between the hippocampus and multiple nodes in rats with suppressed RSCd. **(E)** The links that changed significantly in metabolic connectivity between nodes of rat brain with suppressed RSCd. MC, metabolic connectivity. RSCd vehicle group *n* = 24, RSCd inhibitor group *n* = 22. *p* < 0.05 was considered statistically significant.

**FIGURE 5:**
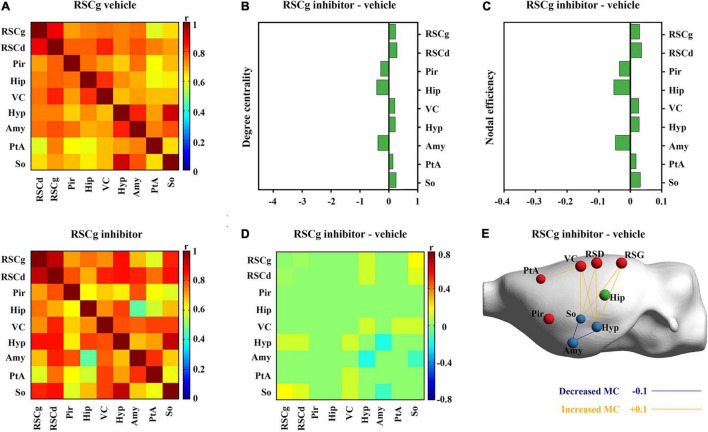
Effects of suppressed RSCg on its core metabolic networks during CFC. **(A)** Core metabolic network of RSCg vehicle and inhibitor groups during CFC. **(B)** Degree centrality and **(C)** nodal efficiency of nodes in RSCg core network do not alter significantly in rats with suppressed RSCg. **(D)** 10,000 permutation tests reveal that the metabolic correlations of the hippocampus with other nodes also do not alter significantly in rats with suppressed RSCg. **(E)** The links that changed significantly in metabolic connectivity between nodes of rat brain with suppressed RSCd. MC, metabolic connectivity. RSCg vehicle group *n* = 35, RSCg inhibitor group *n* = 32. *p* < 0.05 was considered statistically significant.

Then, we examined the differences in properties of core networks among independent groups under different treatments. The RSCd and RSCg, respectively, served as seed regions to screen nodes that were highly metabolically relevant to them (Supplementary Table 3). The members of RSCd core network during CFC include RSCd, RSCg, Pir, Hip, VC, hypothalamus (Hyp), superior colliculus (SC), Amy, parietal association cortex (PtA), and posterior parietal cortex (PPC) (Figure 4A), while the nodes of RSCg core network were RSCg, RSCd, Pir, Hip, VC, Hyp, Amy, PtA, and supraoptic region (So) (Figure 5A).

Nodal graph theoretic measures showed a significant reduction in hippocampal degree centrality (permutation test; Figure 4B) and nodal efficiency (permutation test; Figure 4C) with suppressed RSCd during CFC, whereas the above decrease was not observed upon suppressed RSCg (permutation test; Figures 5B,C).

By comparing the metabolic connectivity of the RSCd core network during CFC between the vehicle and inhibitor groups, a significant decrease in multiple connections associated with hippocampus was observed in the RSCd inhibition group, specifically, the connections Hip–RSCd, Hip–RSCg, Hip–Amy, and Hip–Pir (*p* < 0.05; permutation test; Figures 4D,E). However, metabolic connectivity of hippocampus was not observed significant reduction in the core network of RSCg inhibitor group (*p* < 0.05; permutation test; Figures 5D,E).

## Discussion

The present study examined the effects of RSCd and RSCg on the formation of contextual fear memory in rats and explored their underlying metabolic network mechanisms. Behaviorally, rats with disturbed RSCd showed 5-h and 24-h retrieval impairments, while those with disturbed RSCg did not. Network node degree centrality analysis revealed that both RSCd and RSCg, together with several key structures related to memory and spatial cognition, play hub roles in brain-wide metabolic network during CFC. However, rats with disturbed RSCd showed significantly reduced network connectivity of the hippocampal–amygdalar circuit, while subjects with disturbed RSCg did not.

Previous studies have demonstrated the RSC, as a whole functional entity investigated, is necessary in both formation and retrieval of contextual fear memory (Corcoran et al., 2011; Cowansage et al., 2014). Our findings also seem to be consistent with these reports, rats with disturbed RSCd showing impaired 5-h and 24-h retrieval. More importantly, our findings show that RSCd and RSCg, the major two main constituents of RSC, differ in the effects of rats on CFC behavior. The 5-h and 24-h tests were used to examine the results of memory formation and recent retrieval. For animals with pharmacologically inactivated RSCd before the 5-h test, their freezing periods were significantly decreased than those of controls and animals with suppressed RSCg. For 24-h tests without pharmacological interference, which means that neural correlates for retrieval should not be disturbed, animals also showed a significantly reduced freezing level. These results implicated that memory impairment might be contributed to the disturbed memory information process, not the retrieval phase. While animals with suppressed RSCg before conditioning, neither the 5-h nor 24-h test showed memory impairments. These results suggested that the RSC might mainly rely on the RSCd, not RSCg, to play its role in the contextual fear memory formation process.

The brain-wide metabolic network constructed for control rats showed the neural collections involved in the processes of memory formation. Particularly, some structures viewed as high degree nodes (or hubs) with greater connectivity were identified, which were thought to exert greater effect on network function (Vetere et al., 2017). These regions included the hippocampus, amygdala, RSC (RSCd and RSCg), and VC, happening to be the key brain sites involved in episodic memory and spatial cognition processes. Notably, RSC was one of the most prominent sites, suggesting its global importance and supporting the notion that the RSC might be a pivotal hub of the whole brain (Vann et al., 2009). The very richness of structural connections with other regions provides the basis for the role of RSC in a whole brain range. In both primates and rodents, extensive connections of RSC with numerous other cortices and subcortical nuclei, especially with thalamus, hippocampus, and forebrain, make its potential roles in various memory-related cognition tasks (Vangroen and Wyss, 1990; Kobayashi and Amaral, 2000, 2003, 2007; van Groen and Wyss, 2003). After pharmacologically inactivated, regions influenced by disturbed RSCg and RSCd are different. Under disturbed RSCd condition, a significantly decreased nodal degree occurred in the Pir, the hippocampus, the amygdala, and RSCg, while only the hippocampal degree was decreased in the RSCg situation. This finding suggested that the RSCd influenced the key memory-related structures, hippocampus and amygdala, more significantly than the RSCg. Moreover, disturbed RSCd affected RSCg, not vice versa, suggesting that there was a possible directly functional dependency of RSCg on RSCd during encoding processing of contextual conditioning, given their structural coupling.

Furthermore, the analysis for the core metabolic networks involved in RSCd/RSCg during CFC gave more specifically metabolic connectivity insight to the mechanism by which they play roles through interactions with other structures. Regions with high connectivity with RSCd and RSCg, respectively, are almost the same ones, including the Pir, hippocampus, amygdala, VC, and hypothalamus, and they largely overlap with the hub regions of brain-wide network. However, under pharmacological inference conditions, their effect on network properties of other regions differed significantly. Disturbed RSCd caused a decreased connecting strength between itself and the hippocampus, hippocampus and the Pir, and hippocampus and amygdala nuclei. While in the disturbed RSCg situation, the decrease in connecting strength occurred only between the hypothalamus and the amygdala nuclei, and the supraoptic region and the amygdala nuclei. The hippocampal–amygdalar system is viewed as one core mechanism for the information of contextual fear memory, in which the amygdala nuclei is responsible for coupling of conditioning stimuli and unconditioning stimuli and the hippocampus is engaged in encoding and recent storage processing (Ciocchi et al., 2010; Fanselow and Dong, 2010; Strange et al., 2014). Decreased connectivity between these two structures would directly lead to the failure of contextual fear memory formation. One possible mechanism is that, contrasted to RSCg, the RSCd is particularly involved in spatial information processing associated with the environment due to its heavy connection with the VC (Vogt and Miller, 1983). Because of the possible perturbed information flow of contextual information, the hippocampal–amygdala memory formation circuit was not evoked.

Another notable finding is that the disturbed RSCd also reduced the connectivity between the hippocampus and the Pir, while the RSCg did not. In animals, contrasted to human beings, contextual memory for environmental odors plays a vital role in their life and regulates many behaviors crucial to living, in which the Pir/olfactory cortex are always involved by their direct connections with the hippocampus and the amygdala nuclei (Chu and Downes, 2002; Willander and Larsson, 2007). Whether this olfactory learning mechanism is generally engaged in contextual memory or only triggered under certain specific conditions needs further investigation. Taken together, our findings suggest that the RSCd might play its role in the formation of contextual memory by triggering the evocation of the hippocampal–amygdalar system.

In summary, the present study demonstrated the rat RSC might play its role in contextual fear memory formation processes mainly relying on its RSCd constituent rather than the RSCg part, which might be performed by calling the hippocampal–amygdala system. These findings may also provide a useful target for pharmacotherapeutic treatments of some disorders with retrograde amnesia syndrome as well as PTSD with persistent fear. And metabolic network approaches provide an efficient means by which concerning neural correlates under particular tasks can be investigated *in vivo*.

## Data Availability Statement

The raw data supporting the conclusions of this study are available on request to the corresponding authors.

## Ethics Statement

The animal study was reviewed and approved by all protocols were approved by the Institutional Animal Care and Use Committee of Kunming Institute of Zoology, Chinese Academy of Sciences (ID: SMKX-20190820-195).

## Author Contributions

T-TP and HL: conceptualization and writing, review, and editing. T-TP, CL, and Q-XZ: investigation. T-TP, B-BN, T-HZ, WZ, and S-LZ: software and formal analysis. G-HZ, LX, and B-BN: resources. T-TP, CL, and HL: writing – original draft. LX, G-HZ, and D-ML: supervision. LX, B-BN, Q-XZ, and T-HZ: funding acquisition. All authors contributed to the article and approved the submitted version.

## Conflict of Interest

The authors declare that the research was conducted in the absence of any commercial or financial relationships that could be construed as a potential conflict of interest.

## Publisher’s Note

All claims expressed in this article are solely those of the authors and do not necessarily represent those of their affiliated organizations, or those of the publisher, the editors and the reviewers. Any product that may be evaluated in this article, or claim that may be made by its manufacturer, is not guaranteed or endorsed by the publisher.
