# Supplementary material for: Retrosplenial Cortex Effects Contextual Fear Formation Relying on Dysgranular Constituent in Rats
Source: Front Neurosci. 2022 May 3;16:886858. doi: 10.3389/fnins.2022.886858 (PMC9112855; doi:10.3389/fnins.2022.886858)
Supplement: Supplementary file 1 [file Data_Sheet_1.docx]

Supplementary Material

## Supplementary Figures


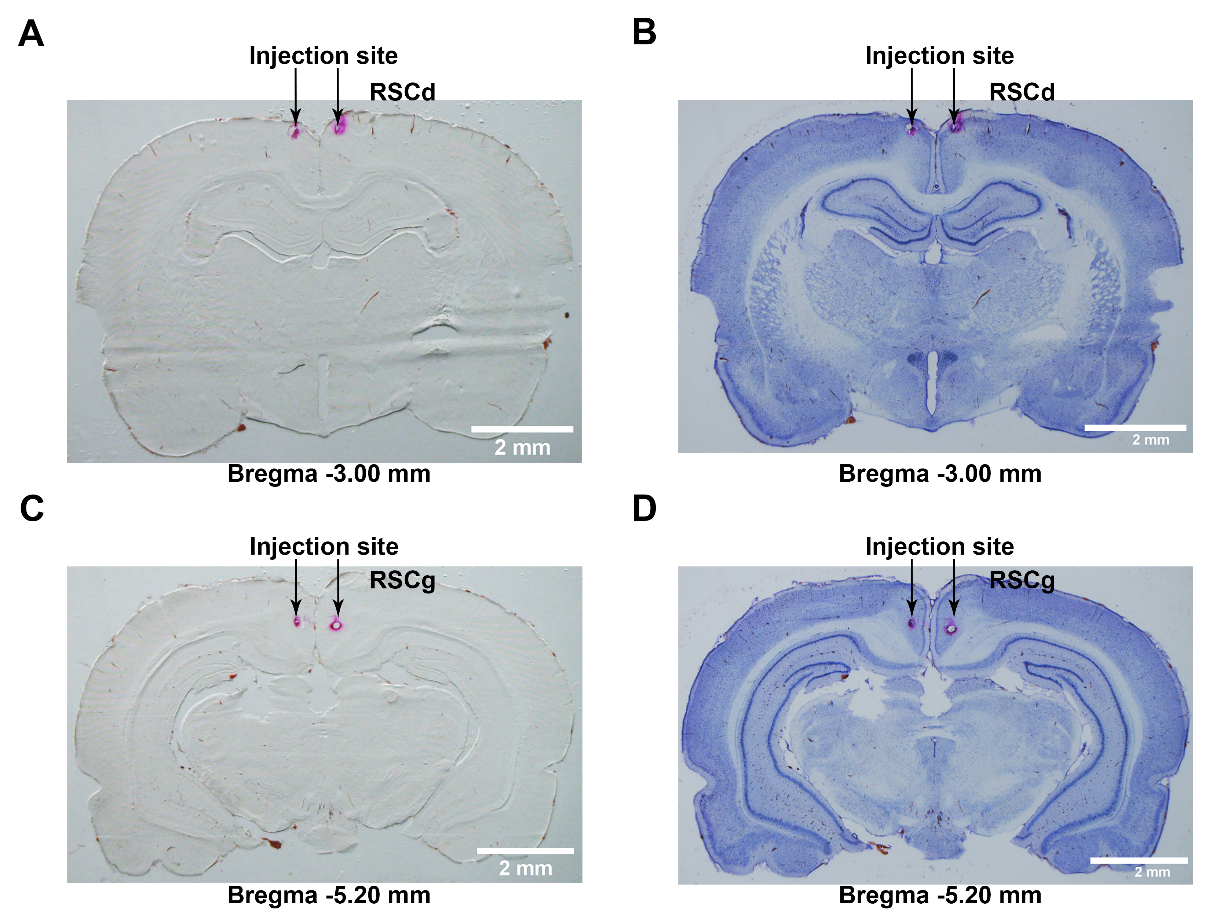


**Figure S1 Characterization of solution injection sites and infiltration range.** Bilateral injections of 1 μL Rose Bengal to characterize the infiltration range of the vehicle/inhibition. Brain section (A) and Nissl staining (B) for RSCd, and brain section (C) and Nissl staining (D) for RSCg.

**Method:** A mixture of inhibitor and Rose Bengal was used to determine the infiltration range of the solution. After 30 min of bilateral injection of 1 μL inhibitor/Rose Bengal mixture into the target brain region, rats were anesthetized with sodium pentobarbital and transcardially perfused with phosphate buffer solution (PBS) followed by 4% paraformaldehyde (PFA). Isolated brains were post fixed in 4% PFA overnight, and dehydrated in 30% sucrose in PBS. The brains were then sectioned (40 μm thick coronal sections) using a vibratome. Subsequently, brain slices were subjected to different concentrations of ethanol gradient dehydration and Nissl staining.


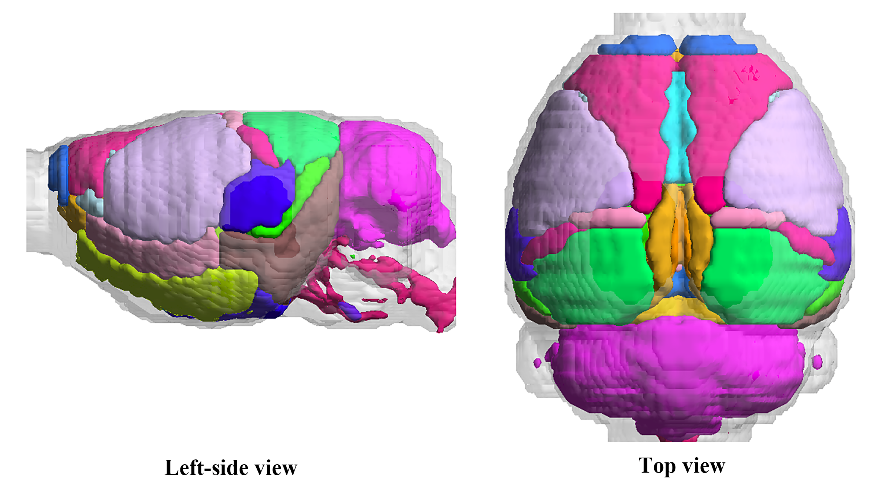


Figure S2 Schematic representation of the location of the 39 brain regions defined as brain nodes, with different colors characterizing different brain regions.

# Supplementary Tables

**Table S1.** Detailed information about the 39 brain structures in rat used for network construction.

| Brain regions | Abbreviations | Paxinos Coordinates (x,y,z) |
| --- | --- | --- |
| Cingulate cortex | Cg | (± 0.80, 1.80, 1.08) |
| Frontal association cortex | FrA | (± 1.69, 3.12, 5.64) |
| Frontal cortex area 3 | Fr3 | (± 4.20, 4.15, 3.72) |
| Infralimbic cortex | IL | (± 0.50, 4.85, 3.00) |
| Insular cortex | Ins | (± 5.60, 6.26, 0.36) |
| Motor cortex | MC | (± 2.23, 1.69, 2.28) |
| Entorhinal cortex | Ent | (± 5.96, 6.57, -7.08) |
| Orbital cortex | Orb | (± 1.60, 4.60, 4.68) |
| Parietal association cortex | PtA | (± 3.15, 1.49, -3.72) |
| Posterior parietal cortex | PPC | (± 5.71, 2.39, -4.92) |
| Piriform cortex | Pir | (± 5.45, 8.80, -0.96) |
| Prelimbic cortex | PrL | (± 0.75, 3.80, 3.72) |
| Sensory cortex | SenC | (± 5.11, 3.12, -0.60) |
| Temporal association cortex | TAC | (± 6.65, 4.73, -7.08) |
| Visual cortex | VC | (± 3.97, 1.82, -6.84) |
| Retrosplenial dysgranular cortex | RSCd | (± 0.60, 1.00, -3.00) |
| Retrosplenial granular cortex | RSCg | (± 0.40, 2.00, -5.20) |
| Auditory cortex | AuC | (± 6.99, 4.62, -4.92) |
| Hippocampus | Hip | (± 4.89, 5.06, -5.40) |
| Supraoptic region | So | (± 0.96, 9.79, -1.20) |
| Thalamus lateral nucleus group | laTh | (± 3.15, 6.12, -3.36) |
| Thalamus medial nucleus group | meTh | (± 0.62, 5.72, -3.48) |
| Thalamus midline nucleus group | miTh | (± 1.22, 7.26, -3.48) |
| Thalamus anterior nucleus group | anTh | (± 0.35, 5.93, -0.12) |
| Inferior colliculus | IC | (± 2.03, 4.35, -8.52) |
| Periaqueductal gray matter | PAG | (± 0.78, 5.60, -7.08) |
| Substantia nigra | SN | (± 2.50, 8.55, -4.68) |
| Superior colliculus | SC | (± 1.60, 4.53, -6.60) |
| Tegmentum of midbrain | Tg | (± 0.95, 6.80, -8.52) |
| Accumbens nucleus | NAc | (± 1.50, 6.82, 1.80) |
| Amygdala | Amy | (± 4.34, 9.18, -3.24) |
| Bed nucleus of stria terminalis | BNST | (± 2.60, 4.95, -1.56) |
| Capsule | Cap | (± 2.15, 2.95, -0.48) |
| Interstitial nucleus | In | (± 3.25, 8.19, -0.24) |
| Red nucleus | RN | (± 1.25, 7.43, -5.64) |
| Septal area | SA | (± 0.61, 5.80, 1.80) |
| Striatum | Str | (± 2.94, 5.93, -0.12) |
| Hypothalamus | Hyp | (± 1.86, 8.50, -1.92) |
| Cerebellum | Cb | (± 2.85, 2.95, -12.12) |

**Table S2.** Nodal degree and Z-score of rat brain-wide metabolic network in four independent groups.

| Node | RSCd group | | | | RSCg group | | | |
| --- | --- | --- | --- | --- | --- | --- | --- | --- |
|  | Vehicle | | Inhibitor | | Vehicle | | Inhibitor | |
|  | Degree | Z-score | Degree | Z-score | Degree | Z-score | Degree | Z-score |
| Cg | 5.08 | -1.29 | 8.73 | 0.10 | 5.25 | -0.94 | 6.54 | -0.45 |
| TAC | 5.27 | -1.18 | 5.43 | -1.23 | 4.31 | -1.48 | 4.72 | -1.52 |
| FrA | 6.11 | -0.69 | 8.23 | -0.10 | 5.77 | -0.63 | 6.67 | -0.38 |
| Fr3 | 5.66 | -0.96 | 6.45 | -0.82 | 5.35 | -0.89 | 5.39 | -1.12 |
| IL | 6.22 | -0.63 | 5.69 | -1.12 | 6.07 | -0.46 | 4.96 | -1.37 |
| Ins | 8.58 | 0.75 | 8.33 | -0.06 | 8.08 | 0.72 | 7.38 | 0.04 |
| RSCd | 9.63 | **1.36** | 10.68 | 0.89 | 9.28 | **1.42** | 9.74 | **1.42** |
| RSCg | 9.53 | **1.30** | 10.83 | 0.95 | 8.95 | **1.23** | 9.01 | 0.99 |
| Orb | 7.84 | 0.32 | 6.17 | -0.93 | 6.98 | 0.08 | 6.39 | -0.54 |
| PtA | 8.61 | 0.76 | 10.38 | 0.76 | 7.88 | 0.60 | 8.71 | 0.82 |
| PPC | 8.85 | 0.90 | 9.84 | 0.55 | 8.36 | 0.88 | 8.08 | 0.45 |
| PrL | 5.27 | -1.19 | 4.64 | -1.55 | 4.54 | -1.35 | 4.69 | -1.53 |
| Pir | 9.84 | **1.48** | 10.71 | 0.90 | 9.19 | **1.37** | 9.85 | **1.48** |
| Ent | 6.10 | -0.70 | 8.48 | -0.00 | 6.16 | -0.40 | 6.83 | -0.28 |
| MC | 7.14 | -0.09 | 10.34 | 0.75 | 7.43 | 0.34 | 8.22 | 0.53 |
| AuC | 7.49 | 0.11 | 8.35 | -0.05 | 6.55 | -0.17 | 5.76 | -0.91 |
| SenC | 7.87 | 0.33 | 9.92 | 0.58 | 7.87 | 0.60 | 7.56 | 0.15 |
| VC | 9.13 | **1.07** | 11.05 | **1.04** | 8.72 | **1.09** | 9.05 | **1.01** |
| Hip | 9.40 | **1.23** | 9.12 | 0.26 | 9.01 | **1.26** | 8.60 | 0.75 |
| anTh | 4.43 | -1.68 | 6.41 | -0.84 | 4.10 | -1.61 | 6.92 | -0.23 |
| miTh | 5.01 | -1.34 | 2.68 | -2.34 | 4.15 | -1.58 | 3.66 | -2.13 |
| meTh | 6.80 | -0.30 | 7.80 | -0.28 | 5.46 | -0.81 | 5.81 | -0.87 |
| laTh | 4.98 | -1.35 | 3.42 | -2.04 | 4.40 | -1.43 | 3.74 | -2.08 |
| SC | 8.67 | 0.80 | 9.74 | 0.51 | 7.85 | 0.58 | 8.56 | 0.73 |
| IC | 6.20 | -0.64 | 7.94 | -0.22 | 5.39 | -0.85 | 7.78 | 0.28 |
| Tg | 8.76 | 0.85 | 10.93 | 0.99 | 8.37 | 0.89 | 8.53 | 0.71 |
| PAG | 6.01 | -0.75 | 5.15 | -1.34 | 5.71 | -0.67 | 5.57 | -1.02 |
| SN | 7.79 | 0.29 | 10.86 | 0.96 | 7.76 | 0.53 | 8.84 | 0.89 |
| In | 8.51 | 0.71 | 10.78 | 0.93 | 8.24 | 0.81 | 8.67 | 0.80 |
| RN | 7.78 | 0.28 | 10.24 | 0.71 | 6.83 | -0.01 | 8.15 | 0.49 |
| Amy | 9.28 | **1.16** | 11.13 | **1.07** | 8.78 | **1.13** | 9.18 | **1.09** |
| NAc | 9.34 | **1.19** | 11.21 | **1.10** | 8.75 | **1.11** | 9.82 | **1.46** |
| Str | 6.76 | -0.32 | 9.15 | 0.27 | 6.56 | -0.17 | 8.17 | 0.50 |
| BNST | 7.69 | 0.22 | 8.37 | -0.04 | 7.52 | 0.39 | 7.01 | -0.18 |
| Cap | 4.14 | -1.84 | 8.56 | 0.03 | 3.79 | -1.79 | 5.72 | -0.93 |
| SA | 4.05 | -1.90 | 3.24 | -2.11 | 4.04 | -1.64 | 6.17 | -0.67 |
| Hyp | 8.88 | 0.92 | 11.53 | **1.23** | 8.58 | **1.01** | 9.31 | **1.17** |
| So | 8.36 | 0.62 | 11.29 | **1.13** | 8.14 | 0.75 | 8.80 | 0.87 |
| Cb | 7.69 | 0.23 | 6.89 | -0.64 | 7.04 | 0.11 | 6.61 | -0.41 |

**Table S3.** Metabolic connectivity of RSCd/g with other nodes in the brain-wide network during CFC

| Seed ragion | Node | Metabolic connectivity | Seed ragion | Node | Metabolic connectivity |
| --- | --- | --- | --- | --- | --- |
| RSCd | **RSCg** | **0.901617** | RSCg | **RSCd** | **0.886576** |
|  | **Pir** | **0.817298** |  | **Pir** | **0.780488** |
|  | **Hip** | **0.819455** |  | **Hip** | **0.772705** |
|  | **VC** | **0.867762** |  | **VC** | **0.841798** |
|  | **Hyp** | **0.715213** |  | **Hyp** | **0.73976** |
|  | **SC** | **0.722489** |  | **Amy** | **0.794318** |
|  | **Amy** | **0.753057** |  | **PtA** | **0.734883** |
|  | **PtA** | **0.754112** |  | **So** | **0.708528** |
|  | **PPC** | **0.7689** |  | PPC | 0.695774 |
|  | Fr3 | 0.054793 |  | Fr3 | 0.204476 |
|  | PrL | 0.192109 |  | PrL | 0.23943 |
|  | SenC | 0.59968 |  | SenC | 0.627918 |
|  | TAC | 0.399485 |  | TAC | 0.31323 |
|  | IL | 0.67429 |  | IL | 0.656154 |
|  | Cg | 0.06598 |  | Cg | 0.204486 |
|  | FrA | 0.08799 |  | FrA | 0.143468 |
|  | AuC | 0.674955 |  | AuC | 0.608188 |
|  | Ins | 0.64413 |  | Ins | 0.576654 |
|  | MC | 0.415769 |  | MC | 0.551029 |
|  | Ent | 0.358733 |  | Ent | 0.370685 |
|  | Orb | 0.486297 |  | Orb | 0.372399 |
|  | laTh | 0.264579 |  | laTh | 0.247527 |
|  | meTh | 0.407133 |  | meTh | 0.308037 |
|  | miTh | 0.220907 |  | miTh | 0.046971 |
|  | anTh | 0.198981 |  | anTh | 0.181483 |
|  | PAG | 0.372789 |  | PAG | 0.300119 |
|  | SN | 0.614936 |  | SN | 0.622857 |
|  | In | 0.653254 |  | In | 0.627448 |
|  | Tg | 0.697206 |  | Tg | 0.682093 |
|  | RN | 0.563144 |  | RN | 0.454509 |
|  | IC | 0.355258 |  | IC | 0.302447 |
|  | Str | 0.492331 |  | Str | 0.48285 |
|  | BNST | 0.461996 |  | BNST | 0.486059 |
|  | Cap | 0.194767 |  | Cap | 0.19872 |
|  | SA | 0.173166 |  | SA | 0.135404 |
|  | NAc | 0.699162 |  | NAc | 0.627245 |
|  | So | 0.650237 |  | SC | 0.661799 |
|  | Cb | 0.468736 |  | Cb | 0.379767 |
